# ROS Generation in Microglia: Understanding Oxidative Stress and Inflammation in Neurodegenerative Disease

**DOI:** 10.3390/antiox9080743

**Published:** 2020-08-13

**Authors:** Dominic S. A. Simpson, Peter L. Oliver

**Affiliations:** 1 Mammalian Genetics Unit, MRC Harwell Institute, Harwell, Oxfordshire OX11 0RD, UK; d.simpson@har.mrc.ac.uk; 2 Department of Physiology, Anatomy and Genetics, University of Oxford, Parks Road, Oxford OX1 3PT, UK

**Keywords:** neuroinflammation, Alzheimer’s disease, microglia, oxidative stress, neurodegeneration, NADPH oxidase (NOX), lipid droplets

## Abstract

Neurodegenerative disorders, such as Alzheimer’s disease, are a global public health burden with poorly understood aetiology. Neuroinflammation and oxidative stress (OS) are undoubtedly hallmarks of neurodegeneration, contributing to disease progression. Protein aggregation and neuronal damage result in the activation of disease-associated microglia (DAM) via damage-associated molecular patterns (DAMPs). DAM facilitate persistent inflammation and reactive oxygen species (ROS) generation. However, the molecular mechanisms linking DAM activation and OS have not been well-defined; thus targeting these cells for clinical benefit has not been possible. In microglia, ROS are generated primarily by NADPH oxidase 2 (NOX2) and activation of NOX2 in DAM is associated with DAMP signalling, inflammation and amyloid plaque deposition, especially in the cerebrovasculature. Additionally, ROS originating from both NOX and the mitochondria may act as second messengers to propagate immune activation; thus intracellular ROS signalling may underlie excessive inflammation and OS. Targeting key kinases in the inflammatory response could cease inflammation and promote tissue repair. Expression of antioxidant proteins in microglia, such as NADPH dehydrogenase 1 (NQO1), is promoted by transcription factor Nrf2, which functions to control inflammation and limit OS. Lipid droplet accumulating microglia (LDAM) may also represent a double-edged sword in neurodegenerative disease by sequestering peroxidised lipids in non-pathological ageing but becoming dysregulated and pro-inflammatory in disease. We suggest that future studies should focus on targeted manipulation of NOX in the microglia to understand the molecular mechanisms driving inflammatory-related NOX activation. Finally, we discuss recent evidence that therapeutic target identification should be unbiased and founded on relevant pathophysiological assays to facilitate the discovery of translatable antioxidant and anti-inflammatory therapeutics.

## 1. Introduction

### 1.1. Neurodegenerative Disease Is a Global Public Health Challenge

Neurodegenerative diseases are characterised by an excessive and pathological loss of neurones that result in dementia, cognitive impairment, perturbed motor control and, ultimately, death. Due to their debilitating nature and lack of any effective treatment, neurodegenerative diseases are a growing economic burden to society [1]. Combine this unmet clinical need with the global ageing population and neurodegenerative disease is a significant challenge faced by public health resources worldwide [2,3,4]. The global cost of dementia is expected to reach over $2 trillion by 2030 and in the UK, the total spent on dementia care already exceeds that of cancer and chronic heart disease combined [5,6]. Therefore, there is an urgent need to identify key molecular pathways contributing to the aetiology of pathological neuronal loss as this will facilitate the development of therapeutic approaches to combat this global health challenge.

### 1.2. Microglial Activation and Oxidative Stress Are Hallmarks of Neurodegenerative Disease

In the past decade, published research into neuroinflammation has been exponential; a PubMed search for “neuroinflammation AND neurodegeneration” indicates just 87 results in 2009 compared to 710 in 2019. The brain, once considered an “immune-privileged” organ is now understood to be a hub of neuroimmune and glial-lymphatic interactions which are dysregulated in neurodegenerative disease [7,8]. Microglia often referred to as “brain-resident macrophages” are complex and dynamic mediators of neuroinflammation. These phagocytic glia form a heterogeneous network that influences development, maintains homeostasis, surveys the parenchyma and reacts to damage- and pathogen-associated stimuli [9,10]. In response to these stimuli, microglia mediate both protective and deleterious responses to brain insults and so are critical regulators of the immune response in neurodegenerative disease (reviewed in [11]).

Immune involvement in neurodegenerative disease centres on findings that pro-inflammatory microglia are closely associated with protein aggregate pathologies characteristic of most dementias. In Alzheimer’s disease (AD), the most common form of dementia, microglia surround amyloid plaques and respond to amyloid-β with a pro-inflammatory phenotype characterised by cytokine expression (e.g., interleukins (IL) 1β and 6 and tumour necrosis factor α (TNFα)) that may initially limit amyloidosis but likely becomes overzealous and neurotoxic [12,13,14]. Expression of amyloid-scavenging receptors (such as CD36) and amyloid-degrading enzymes decrease as aberrant inflammatory regulation ensues in AD [13]. Characterisation of gene ontology networks in post-mortem AD brains has identified dysregulation of microglia-specific networks as the most strongly correlated to neuropathology [15] and many microglial AD risk-factor genes have been identified [16,17]. These genes include triggering receptor expressed on myeloid cells 2 (*TREM2*), a pro-inflammatory signalling receptor which may bind amyloid-β and ApoE, and progranulin (*PGRN*), a trophic factor and inflammatory regulator [16,17,18,19].

Similarly, activated microglia are spatially correlated with microtubule-associated protein tau (MAPT, tau) pathology and are capable of recognising and clearing tau [20,21]. Further, activated microglia promote tau phosphorylation and aggregation by activation of the NLRP3 (nucleotide binding oligomerisation domain (NOD)-, leucine-rich repeat (LRR)- and pyrin domain-containing protein 3) inflammasome in response to amyloid-β [22,23]. Critically, this identifies a mechanistic link between microglial activation in AD and the development of tau pathology. Microglia also phagocytose α-synuclein, the constituent protein of Lewy bodies in Parkinson’s and Lewy Body dementias, and become pro-inflammatory in the process, expressing cytokines (IL1β, IL6, TNFα), reactive oxygen species (ROS) and prostaglandin synthesising cyclooxygenase 2 (COX2) [24,25,26].

Furthermore, microglia respond to damage-associated molecular patterns (DAMPs)—molecules released from damaged cells—by the production of ROS, which becomes elevated and sustained in disease [27,28,29]. Since oxidative stress is a hallmark of AD, indicated by lipid peroxidation [30,31,32,33], protein oxidation [34,35] and mitochondrial DNA damage [36], microglial ROS likely contribute to oxidative stress associated with neurodegeneration. Thus, understanding how disease-associated microglia (DAM) become dysregulated and contribute to oxidative stress by ROS production is crucial to understanding inflammatory-associated neurodegeneration.

Clearly, microglia play a key role in the pathophysiology of neurodegeneration and understanding their contribution will allow us to harness their protective potential and limit chronic, damaging inflammation. In parallel, it is important to consider that microglia assume a range of phenotypes in response to homeostatic and damage-associated stimuli [9,10,37]. In doing so, microglia mediate the balance between beneficial, damage-limiting homeostasis and chronic inflammatory states that underpin pathological neuronal loss. Thus, identifying key molecular targets underlying changes to microglial physiology will be critical to developing neuroimmune modulatory therapies.

### 1.3. The Brain Is Especially Susceptible to Oxidative Stress

ROS are maintained in dynamic equilibrium balanced by ROS-generating cellular processes and antioxidant defences. Oxidative stress describes the damage that occurs when ROS overwhelm the antioxidant defence systems. This phenomenon is a consequence of disrupted homeostasis, such that production of ROS becomes excessive or due to diminution of defence systems [38]. The brain is particularly susceptible to oxidative stress due to a triad of elevated ROS production, modest antioxidant defences and a limited capacity for regeneration [39]. As phagocytes, microglia are capable of mediating an “oxidative/respiratory burst” in response to pathogen- and damage-AMPs, including aggregated protein and cellular debris [40,41]. Thus, ROS are an important part of the arsenal employed by microglia in tissue defence. However, since there is a strong correlation between immune activation and oxidative damage in neurodegenerative disease [42,43,44], it is hypothesised that elevated and dysregulated ROS production from DAM contributes to oxidative stress, leading to neuronal death.

There are several important mechanisms by which oxidative stress and neuroinflammation are intricately linked. They can be classified in three ways: (1) inflammatory-related ROS production, (2) inflammation induced by ROS as secondary messengers and (3) lipid droplet accumulation in microglia.

## 2. Inflammatory-Related ROS Production—NOX Mediates Production of ROS in Microglia

### 2.1. NOX Are a Family of Enzyme Subunits that Mediate Deliberate Production of ROS in Inflammation

To understand the dysregulation of ROS production associated with neuroinflammation, it is critical to understand the source of ROS in the neuroimmune system and its physiological regulation. NADPH oxidase (NOX) is a group of seven ROS-generating enzymes: NOX1 to 5 and dual oxidase (DUOX) 1 and 2. NOX is best characterised in peripheral phagocytes (macrophages and neutrophils), where NOX2 or Phox (phagocyte NOX, gp91^phox^) mediates the oxidative burst during phagocytosis or in response to pro-inflammatory signals, such as IFNγ [45]. Loss-of-function mutations in NOX2 result in chronic granulomatous disease, a rare syndrome described in 1957 and characterised by recurrent infections caused by impaired phagocytosis [46,47,48]. Studies of phagocytes from these patients resulted in the identification, characterisation [49,50,51] and eventual cloning of NOX2 in 1986 [52,53]. The advent of PCR-based cloning, along with sensitive ROS assays in the 1990s, allowed for the characterisation of other NOX isoforms that often generate ROS in specialised cell types. For example, NOX1 was identified in smooth muscle cells and is activated in response to cell-growth signals, whereas NOX4 acts as a renal oxygen sensor [54,55]. Upon activation by the assembly of multiple subunits, NOX enzymes generate the superoxide radical (O_2_^•–^). The exception is NOX4, which is constitutively active, independent of co-expression with regulatory subunits, and mostly produces H_2_O_2_ [56,57]. Unlike mitochondrial ROS, which are by-products of oxidative phosphorylation, ROS generated from NOX are deliberate and part of the antimicrobial armoury of phagocytes [58].

### 2.2. NOX Enzymes Can Be Subgrouped Based on Their Homology to gp91^phox^ and Regulation of Activity

There are three subgroups of NOX according to their regulation of activity and homology to the membrane-bound component, gp91^phox^ (NOX2) [59]. NOX1, 2 and 3 are similar in structure and size and are thus sub-classified as NOX2-like. The activity of these enzymes depends on the assembly of several cytosolic regulatory subunits. NOX4 also shares gp91^phox^ homology; however it is active when expressed in absence of cytosolic regulatory subunits and functions as a renal oxygen sensor [56,57]; indeed NOX4 can generate H_2_O_2_ as a function of partial pressure of O_2_ over a wide range, allowing it to rapidly detect and respond to changes in O_2_ availability [55]. NOX5 and DUOX1/2 comprise the third group and display functional additions to gp91^phox^. NOX5, not expressed in rodents, contains the membrane-bound gp91^phox^ domain plus an additional N-terminal calmodulin-like moiety. DUOX1 and DUOX2 build on the NOX5 structure, with an added N-terminal peroxidase domain. The peroxidase domain is predicted to face the extracellular milieu and may catalyse the oxidation of extracellular cofactors by reducing self-generated O_2_^•–^ to H_2_O_2_, hence the dual oxidase function [60] (NOX biology is reviewed more comprehensively by Lambeth [59]).

### 2.3. Phagocytic NOX (NOX1-3) Are Regulated by the Assembly of Cytosolic Subunits

**Figure 1:**
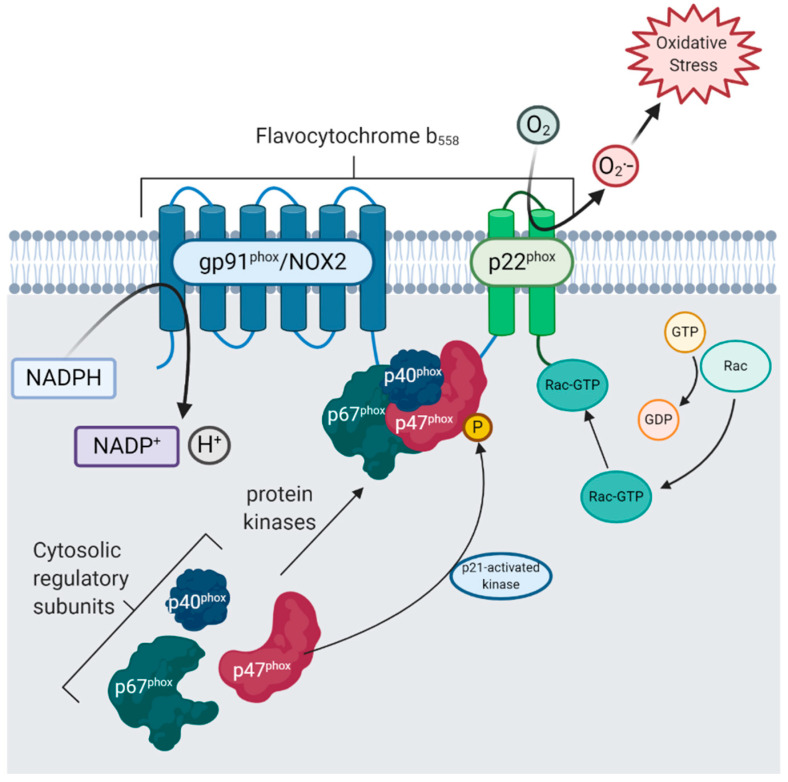
NADPH oxidase 2 (NOX2) is activated by translocation of cytosolic subunits to the membrane. gp91^phox^ and p22^phox^ are membrane-bound components of NOX2 that together form the flavocytochrome b_558_. Upon activation signalling by pro-inflammatory stimuli, e.g., interferon-γ, protein kinases activate the cytosolic regulatory subunits: p40^phox^, p47^phox^ and p67^phox^. Phosphorylation of p47^phox^ by p21-activated kinase is thought to be the rate-limiting step in NOX2 activation [62]. The small GTPase Rac1/2 is also activated and translocated to the flavocytochrome. Upon activation, NOX2 catalyses the production of the superoxide radical (O_2_^•–^) from molecular oxygen (O_2_) by oxidation of nicotinamide adenine dinucleotide phosphate (NADPH) to form NADP^+^ and H^+^.

The canonical phagocytic NOXs (NOX1-3) oxidise cytosolic NADPH across the plasma membrane to generate O_2_^•−^, which can be further reduced to H_2_O_2_. They are structurally similar to NOX2, which is accompanied by regulatory subunits, namely p22^phox^, p47^phox^, p40^phox^ and p67^phox^ and the small GTPase, Rac1/2. p22^phox^ is membrane-bound and associated with gp91^phox^, which together form flavocytochrome b_558_ (Figure 1). Rac1/2, p40^phox^, p47^phox^ p67^phox^ are cytosolic subunits which translocate to the membrane upon activation signalling to assemble the active enzyme complex with the same membrane-bound flavocytochrome [61]. Thus, the activity of phagocytic NOX is regulated by the membrane translocation of these cytosolic subunits. The rate-limiting step of NOX2 activation is hypothesised to be serine-threonine phosphorylation of p47^phox^ by p21 (cdc42/Rac1)-activated kinase-1 [62]. It is possible other relevant serine-threonine kinases, including the mitogen-activated protein kinase (MAPK) family and protein kinase C, are capable of catalysing NOX2 assembly. Since these pathways converge on pro-inflammatory signalling in DAM, NOX2 activation may be an important pro-inflammatory mechanism in microglia.

## 3. Expression and Regulation of NOX in Microglia

The cellular expression of NOX enzymes throughout the CNS has been thoroughly reviewed, however, the precise localisation of different NOX isoforms requires further examination [63]. Induced expression of NOX1, NOX2, NOX3 and NOX4 has been reported in neurons in pathological conditions such as mechanical injury, mostly by immunofluorescent detection [64,65]. Neuronal expression of NOX does not appear to be constitutive and the validation of detection methods used by stringent controls and transcriptomic corroboration is inadequate [63,66]. Robust and constitutive expression of NOX in microglia has been demonstrated and is the focus of this section.

Since NOX activation relies upon the assembly of multiple subunits, the primary activity regulation is likely dependent on protein kinetics, except for the constitutively active NOX4 which is regulated at the transcriptional level [56,67]. Indeed, the expression of cytosolic regulatory subunits of NOX2 (p67^phox^, p47^phox^ and p40^phox^) is positively correlated with disease progression and oxidative stress in AD, whilst expression of membrane-bound flavocytochrome b_558_ subunits (gp91^phox^ and p22^phox^) is stable [68]. This suggests greater availability of cytosolic regulatory subunits contributes to excessive NOX2 activation in AD. However, to deduce the relative importance of NOX isoforms in neuroinflammation, it is important to consider which isoforms are expressed in microglia, hence responsible for ROS generation. Furthermore, the maximum capacity of microglia to produce ROS will be determined, at least in part, by the expression level of NOX isoforms.

### Microglia Express NOX2 and NOX4

Whilst peripheral macrophages and microglia share origin in the yolk sac, microglia are an ontogenetically distinct, locally maintained cell population [69,70] and so NOX expression in microglia may not exactly mirror that of their macrophage relatives. Despite this, single-cell (sc)RNA-seq has determined that NOX2 (*CYBB*) is the most highly expressed NOX isotype transcript in human and mouse brain microglia, concurrent with expression in peripheral macrophages [71,72,73]. NOX4 has the second-highest transcript expression in microglia, albeit at a much lower level [72,73]. Quantitative proteomic analysis of NOX expression to corroborate transcriptional studies is limited by a lack of specific antibodies and stringent controls. However, in situ hybridisation studies in zebrafish have indicated varying expression of NOX isoforms throughout the development of the CNS, with robust and stable expression of NOX2 throughout embryogenesis [74].

NOX4 is expressed in the mammalian brain, however specific proteomic expression in microglia is unknown. In situ hybridisation has demonstrated upregulated and persistent cortical NOX4 expression in response to cerebral ischaemia [75] and manipulation of expression has identified it as a key regulator of ROS generation in human microglial cell line clone 3 [76]. Knockdown of NOX4 in these cells suppresses both ROS and interleukin (IL)6 production, confirming a link between NOX4 activation and pro-inflammatory cytokine expression. Interestingly, however, this particular cell line does not express NOX2; this calls into question the validity of the model since NOX2 is the most highly expressed isotype in both human and murine microglia [72,73]. Nonetheless, NOX4 activation in disease conditions, such as cerebral ischaemia, may result in ROS production and the generation of IL6 in mouse microglia.

NOX1 transcript and protein expression have been described in microglia isolated from Cx3cr1-enhanced green fluorescent protein (EGFP) reporter mice [77], although expression has not been demonstrated in the mouse brain elsewhere, to our knowledge [65]. NOX1 expression may be an artefact of the Cx3cr1-EGFP reporter line, since Cx3cr1 is a signature microglial gene and haploinsufficiency has wide-reaching effects on gene expression of microglia, specifically encouraging a prematurely aged transcriptome [78,79,80]. Nevertheless, transcriptional expression of NOX1 could not be corroborated in RNAseq of Cx3xr1 haploinsufficient mice [78]. It is necessary to determine whether NOX1 is indeed expressed in microglia, and in which circumstances, to understand its contribution to inflammation and oxidative stress. The physiological and transcriptional heterogeneity of microglia throughout life, differences in brain regions and disease have become apparent [81,82] and it may be possible that NOX1 is expressed in different stages of development, in ageing or certain pathological settings.

In summary, expression and activation of NOX isoforms in the central nervous system, with relevance to spatial and temporal specificity, requires further investigation. For example, NOX4 is induced in ischemia but cell-type expression is unknown [75] and the expression of NOX1 in microglia is likely an artefact of disruption of the Cx3cr1 locus [77,78]. NOX2 is expressed robustly in both human and murine microglia and the adult CNS and throughout development, indicating a strong evolutionary requirement for its expression in the CNS. Crucially, many studies investigating the cellular expression of NOX enzymes lack stringent controls (i.e., genetic deletion of specific isoforms) to verify the specificity of the reagents used. Ultimately, expression studies of NOX in the brain are lacking and it is important to determine where NOX isoforms are expressed and their response to disease-associated stimuli and in different brain regions as we become increasingly aware of the regional heterogeneity of microglia [81].

## 4. Microglial NOX Is Activated in Inflammation and Neurodegeneration

### 4.1. NOX2 and NOX4 Are Activated by Acute, Pro-Inflammatory Stimulation of Microglia

The robust activation of NOX isoforms in response to acute neuronal injury and chronic inflammation with oxidative damage has been well documented in post-mortem tissue and models of neurodegeneration (reviewed in [83]). The localisation of NOX activity and expression in these studies is key to determine the cellular source of damaging ROS. Further, understanding the relative importance of NOX isoforms in specific cell types will expedite the development of targeted, efficacious therapeutic options.

Unsurprisingly, the classical phagocytic NOX, NOX2, plays a key role in inflammatory-mediated ROS production in microglia. Primary midbrain cultures from NOX2 knockout rats are more resistant to neurotoxicity by synergistic treatment with lipopolysaccharide (LPS) and α-synuclein [84]. This indicates that NOX2 involvement in neuroinflammation may be associated with Parkinson’s disease. Neuronal resistance to toxicity is thought to occur via perturbation of NOX2 in microglia, as LPS and α-synuclein elicit a mitotic, pro-inflammatory and pro-oxidant response in microglia, which is not evident in NOX2 knockout cultures [84]. The specific knockout of NOX2 in microglia would confirm this. There remains a small increase in ROS production in response to LPS and α-synuclein in these NOX2 knockout cultures, likely a result of NOX4, since this isoform is expressed at the next highest level in microglia [72]. NOX2 activity in dopaminergic neurodegeneration is mediated by activation of complement receptor 3 (CR3, aka macrophage-1 antigen (MAC1)), which is a pattern recognition receptor (PRR) comprised of CD11b and CD18 and agonised by DAMPs which contributes to phagocytic activation in response to aggregated protein [85,86]. CR3 stimulation results in extracellular signal-regulated kinase (ERK)-stimulated translocation of p47^phox^ to the plasma membrane indicating NOX2 activation [86].

Immunofluorescence has indicated NOX2 is the most responsive isotype to mechanical brain and spinal cord injury, with elevated expression persisting beyond 28-days post-injury in line with its robust expression [64,87]. Genetic deletion or pharmacological inhibition of NOX2 in a mouse model of traumatic brain injury (TBI) demonstrates attenuation of pro-inflammatory microglial phenotype, indicated by reduced expression of cytokines, including IL1β, IL6 and TNFα, 1-day post-injury [88]. Moreover, the expression of arginine, suppressor of cytokine signalling 3 (SOCS3) and IL1 receptor antagonist (IL1Ra) was increased, implying resolution of inflammation and tissue repair. Functionally, repression of NOX2 encouraged some cognitive improvement, however, no improvement to motor function was seen [83]. This highlights the importance of targeting inflammation at the optimum time-point, to limit tissue damage and promote repair. Nevertheless, data suggest NOX2, along with mediating sustained ROS production, is a driver of excessive inflammation in response to neuronal injury and may impede inflammatory resolution in vivo.

### 4.2. Damage-Associated Molecular Patterns (DAMPs) Originating from Neurons Mediate NOX Activation via Pattern Recognition Receptors CR3 and TLR4

**Figure 2:**
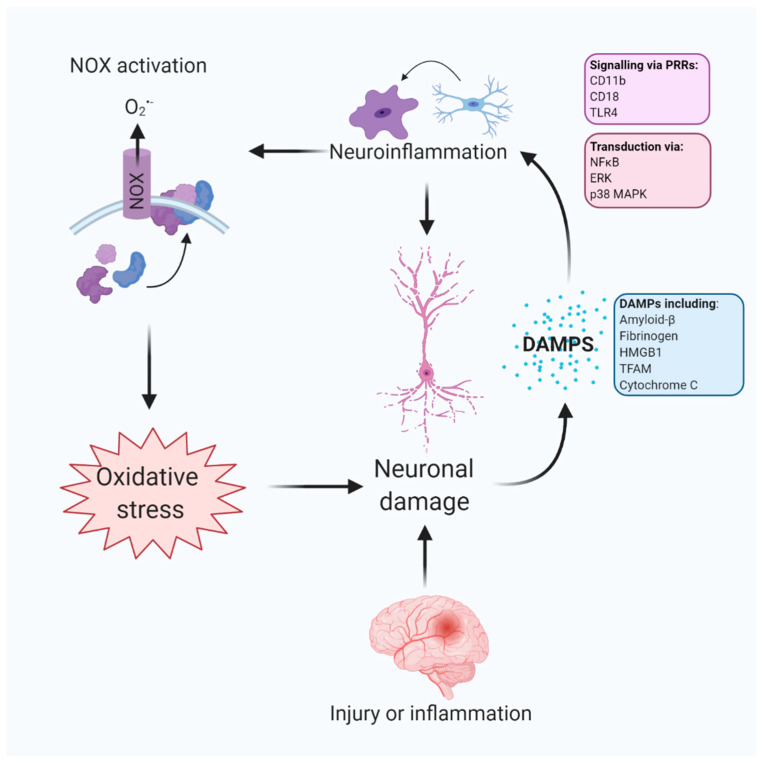
Damage-associated molecular patterns (DAMPs) contribute to neuroinflammation and oxidative stress. DAMPs originate from damaged or dying cells in response to acute injury and neuroinflammation. DAMPs, such as amyloid-β, fibrinogen, high mobility group box 1 (HMGB1), mitochondrial transcription factor A (TFAM) and cytochrome c may contribute to inflammatory-driven phagocytic activation of microglia by signalling on pattern recognition receptors (PRRs), such as complement receptor 3 (CD11b, CD18) and toll-like receptor 4 (TLR4). These signals are transduced in microglia by nuclear factor κB (NFκB) and mitogen-activated protein kinases (MAPKs), such as p38 and extracellular signal-regulated kinase (ERK). Phagocyte activation causes translocation of NADPH oxidase (NOX) subunits to the plasma membrane to activate NOX and drive reactive oxygen species generation, contributing to oxidative stress.

Neuronal damage is an important triggering factor for NOX activation in microglia, likely mediated by DAMPs originating from neurones in both acute injury and chronic neuroinflammation [89,90]. DAMP stimulation of PRRs on microglia, such as CR3 and toll-like receptor 4 (TLR4), mediates activation of pro-inflammatory signalling transducers NLRP3 inflammasome, nuclear factor kappa-light-chain-enhancer of activated B cells (NFκB) and mitogen-activated protein kinases (MAPKs), such as ERK [86,91,92]. Considering CR3 signalling is an essential functional component in synaptic pruning by microglia [93], NOX activation by PRRs is likely a functional inflammatory-related phagocytic response to eliminate dead and dying neurons and aggregated protein, which has been elucidated by 2-photon live imaging [94,95]. Hence, DAMP signalling on these receptors may underpin excessive ROS production by inflammatory-related phagocytic activation and prime a vicious cycle of DAMP-induced neuronal damage by oxidative stress [86,96] (Figure 2).

The specific DAMPs, their receptors and intracellular transduction in microglia leading to NOX activation require further elucidation, despite the role of DAMPs in neuroinflammation being well-characterised (reviewed in [97]). CR3, which is upregulated in human AD-associated microglia, contributes to persistent inflammation, NOX2 activation and O_2_^•-^ release in response to DAMPs, such as amyloid-β [85,98,99,100]. Both in co-culture and in vivo, toxins associated with neurodegeneration, such as 1-methyl-4-phenyl-1,2,3,6-tetrahydropyridine (MPTP), LPS and diesel exhaust particles, cause CR3 stimulation by the DAMP, high mobility group box 1 (HMGB1), which results in activation of NOX2 and production of O_2_^•–^ [101,102,103]. Pharmacological blockade or microglial-specific knockout of TLR4 or CR3 severely attenuates ROS production by NOX2, indicating NOX2 activation is of microglial origin, despite reported expression of NOX2 in neurons [64,102,103]. Furthermore, fibrinogen, a DAMP which is closely associated with cerebral amyloid angiopathy (CAA), induces ROS production by NOX2 in microglia via stimulation of CR3 [104]. Considering HMGB1 is an important stimulus for microglial activation, this protein is a key link between inflammatory activation and oxidative stress and its role in chronic neuroinflammation associated with neurodegeneration should be explored [104]. Understanding the role of other neuroinflammatory-related DAMPs, such as cytochrome c and mitochondrial transcription factor A (TFAM) will aid in understanding the molecular mechanism driving NOX activation in neurodegeneration.

### 4.3. NOX Is Activated in Chronic Disease-Associated Microglia (DAM)—A Focus on Alzheimer’s Disease

#### 4.3.1. Alzheimer’s as a Chronic Inflammatory Disease

Activation of NOX in acute injury and inflammation is clear, however, neurodegeneration is a chronic disease characterised by progressive neuronal loss and more subtle changes to the brain parenchyma that have been described as “the manifestation of a faster ageing process” [105]. Therefore, it is important to describe microglial NOX in this accentuated ageing as well as in response to more acute stimuli. Sporadic, late-onset Alzheimer’s disease (AD) is the most common neurodegenerative disease with an estimated over 50 million cases globally as of 2019 and perhaps the best example of chronic neurodegeneration [106,107]. AD presents with age-related, progressive, neurodegeneration thought to result from toxic aggregation of extracellular amyloid-β and intracellular tau [108]. It has become apparent that targeting idiopathic build-up of amyloid is insufficient to reverse neurodegeneration and cognitive decline associated with the disease [109,110,111]; indeed, this has prompted researchers to reconsider the aetiology of AD. Further investigation of DAM has generated many alternative and complementary hypotheses, including oxidative stress, which may be mediated by NOX activation in DAM [112].

#### 4.3.2. NOX Is Activated in the Human AD Brain

NOX activation is evident in the AD brain. The NOX2 regulatory subunits, p47^phox^ and p67^phox^, are translocated from the cytosolic to the membranous fractions of AD brains, suggesting NOX2 activation [113]. Also, NOX1 and 3 expression is increased in early AD, followed by upregulation of markers of mitochondrial dysfunction [114]. Furthermore, ante-mortem cognitive impairment is correlated with post-mortem determination of NOX activity in the frontal and temporal cortices [68]. Thus, NOX2 activation, along with elevated expression of NOX1 and NOX3, may be directly related to neuronal loss manifesting as cognitive decline in AD. The localisation of isoform activity in the AD brain is unknown, however, NOX2 is highly expressed in microglia [72], therefore it is likely microglial NOX activation accounts for at least part of AD-related NOX activity.

#### 4.3.3. Mammalian Models of AD Indicate NOX Activation Contributes to Neuronal Loss

Evidence from mammalian studies indicates that NOX activation in response to amyloid-β contributes to neurodegeneration and is associated with cerebrovascular pathology. Inhibiting NOX with the naturally occurring compound, apocynin, or genetic deletion of gp91^phox^ in mice (Tg2576) overexpressing mutant amyloid precursor protein (APP), attenuates cerebral amyloid angiopathy (CAA) [115,116]. These findings have been complemented in a humanised APP/PS1 (hAPP/PS1, human mutant APP and presenilin-1) mouse; whilst neurovascular changes were not reported, increased NOX activity and NOX4 expression were age-dependent and NOX activity was, again, strongly correlated with cognitive impairment [117]. Crucially, these data highlight that NOX activation is related to amyloidosis even when mutant human amyloid-processing proteins are expressed at endogenous levels. Indeed, overexpression in classical APP rodent models may explain why cognitive amelioration could not be replicated by apocynin in the Tg19959 mutant [118]. It is now important to determine whether NOX plays a role in cerebrovascular neurodegeneration when amyloid-related transgenes are expressed at endogenous levels. Nonetheless, NOX is activated in in vivo models of AD and offsetting this upregulation may limit neurodegeneration and cognitive impairment.

#### 4.3.4. NOX Is Activated in AD-Associated Microglia

It is noteworthy that the cellular localisation of NOX activation in mammalian models of AD has not been absolutely determined. However, p47^phox^ and p67^phox^ are not expressed in neurons or astrocytes isolated from rats, but cytosolic translocation of both subunits does occur in microglia [113]. This suggests a functional specificity of NOX2 in microglia. Further, microglia in AD brains strongly express gp91^phox^, implying NOX2 activation is localised to microglia in AD [119]. Inhibiting NOX by genetic deletion limits the microglial activation response to LPS and may promote adoption of a healing or inflammation-limiting phenotype, determined by elevated expression of IL4 in p47^phox-/-^ microglia [120]. Furthermore, amyloid-β stimulates NOX2 in rat microglia and peripheral phagocytes, whilst phagocytes isolated from patients with chronic granulomatosis disease (caused by a genetic deficiency of NOX2) do not respond to stimulation with amyloid-β [121]. Studies must now consider human induced pluripotent stem cell (iPSC)-derived microglia and whether these findings translate in cells from healthy and AD individuals. Additionally, human CAA post-mortem capillaries with amyloid-β load are surrounded by NOX2-positive microglia [122,123]. This indicates that amyloid-β recognition by receptors expressed as part of the microglial sensome, which includes PRRs such as CR3, may involve activation of NOX2 as a response to local amyloid-β accumulation [124]. Indeed, Aβ-42 induces NOX2 expression in microglia and this response is accentuated in aged mice [125]. Furthermore, age-related amyloid deposition, microgliosis and ROS production were dependent on NOX2 expression and findings correlated with post-mortem tissue from young and aged human samples [125]. IL13 may be involved in microglial NOX activation associated with CAA since hippocampal thrombin injection stimulates IL13 production, which leads to microglial NOX2 activation and neurodegeneration in vivo [126]. However, thrombin injection initiates a near-immediate pseudoaneurysm which represents a more acute and overt stimulus for neurodegeneration than the subtle, slow changes involved in AD and amyloidosis. Nevertheless, fibrinogen, a NOX-activating DAMP which is converted to fibrin by thrombin to cause clotting, is a mediator of microglial oxidative stress and neurodegeneration in a 5x familial AD mutations (5xFAD) mouse model [104]. Again, these effects were ameliorated by apocynin, suggesting NOX activation and further supports a role for clot-related factors in microglial NOX activation. Therefore, it is crucial to enhance NOX activation at the cellular level in these in vivo models of AD. Taken together, these findings strongly suggest a specific role for NOX2 activation in microglia associated with cerebral amyloid pathology.

#### 4.3.5. Further Investigation of Microglial NOX Should Focus on Tau Pathology and Cell-Specific Manipulation of NOX

The response of NOX activity to tau, the intracellular partner of amyloid-β in AD, has been relatively understudied and the role of tau in microglial NOX activation remains unassessed. Tau aggregates in neuronal-astrocytic co-cultures stimulate NOX activity and ROS production leading to neuronal death [127]. This suggests that the NOX response to tau may be, at least in part, localised to astrocytes or neurones. Indeed, the role of astrocytes in neuroinflammation and oxidative stress is undoubtedly important but beyond the scope of this review; this particular topic is considered in detail elsewhere [128]. Further studies are required to understand the relationship between microglial NOX activation and tauopathy; this is especially pertinent when considering the increased appreciation of aggregated tau in AD pathology. Tau-driven epigenetic changes, tau seeding activity and variations in post-translational modification of tau may underlie individual heterogeneity in AD progression and understanding how tau contributes to oxidative stress and inflammation is vital [129,130]. Further, there is evidence tauopathy is driven by NLRP3 inflammasome activation in microglia in response to amyloid-β [23]. Considering AD-associated microglia are a vital mediator of tau aggregation, aberrant processing of tau driven by microglia may provide a stimulus for further oxidative stress, perhaps driven by astrocytes [127].

Since NOX expression has been reported in other CNS cell types, including neurons [64] and astrocytes [131], targeted deletion of NOX subunits in microglia, for example, making use of transmembrane protein 119 (Tmem119)-driven cre recombinase—a microglial gene with exceptional specificity—would prove an excellent tool for studying the role of microglial ROS generation in disease settings [132,133]. Not only would this identify the specific role of microglial NOX activity by preserving expression in other cell types but mammalian cross-breeds incorporating AD pathology and NOX deletion would allow for full characterisation of the specific role of microglial NOX in AD.

In summary, elevated NOX activity and expression in DAM plays a critical role in neuronal loss seen in AD as a result of neuroinflammatory responses to amyloid-β, especially related to the cerebrovasculature. The role of microglia in the development of tauopathy in response to amyloid-β has been scrutinised but the role of tau must be considered in efforts to understand the aetiology of AD. Thus, the microglial oxidative stress response to tau must be characterised and understood. Finally, pharmacological studies have indicated that manipulation of the intracellular microglial signalling pathways linked to transforming growth factor β-1 (TGF-β1) receptor [134] and angiotensin II type-2 (AT2) receptor [135] can inhibit NOX activation and drive an inflammatory resolution phenotype by modulating targets such as protein kinase C. Further studies looking to modulate these pathways in vivo, with relevance to AD, are critical for developing effective therapeutics that target ROS production from activated microglia.

## 5. ROS Are Secondary Messengers Activating Pro-inflammatory Pathways in Microglia

**Figure 3:**
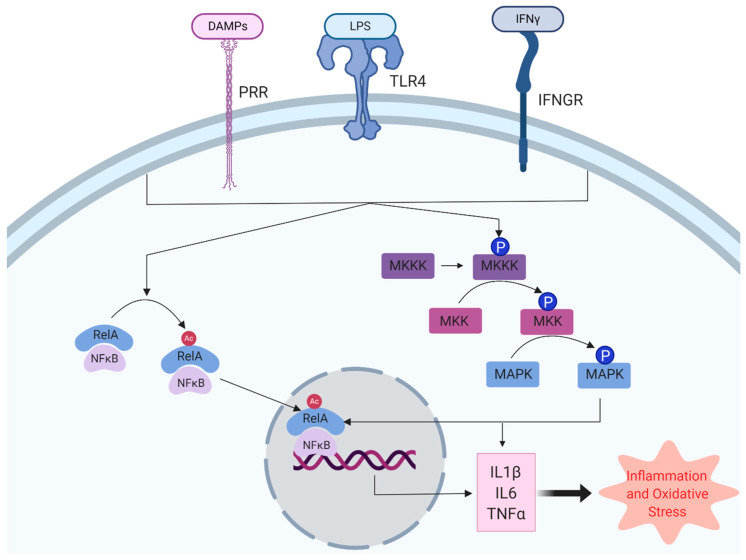
Control of microglial activation by NFκB and MAPKs. Proinflammatory stimulation of microglia is mediated by damage-associated molecular patterns (DAMPs), lipopolysaccharide (LPS) and interferon-gamma at pattern recognition receptors (PRRs), toll-like receptor 4 (TLR4) and interferon-γ receptor, respectively. Signal transduction by nuclear factor kappa-light-chain-enhancer of activated B cells (NFκB) and mitogen-activated protein kinases (MAPKs) results in the upregulation of cytokines, such as interleukin (IL) 1β, IL6 and tumour necrosis factor α (TNFα) which contribute to chronic inflammation and oxidative stress in the CNS.

At physiological concentrations, ROS are vital secondary messengers in multiple cellular processes, including inflammation, cellular growth and differentiation [136,137]. Having considered how immune activation results in ROS production, it is also pertinent to consider how ROS contribute to immune activation in the brain as secondary messengers, especially as this may underlie a reciprocal interaction. Multiple key signalling pathways contribute to microglial immune activation, including NFκB and MAPKs (Figure 3) and there is evidence ROS is an important modulator of the immune response by influencing these cell signalling pathways in microglia.

### 5.1. NFκB, a Master Regulator of Inflammation in Microglia, Is Associated with NOX Expression

Nuclear factor kappa-light-chain-enhancer of activated B cells (NFκB) is a master transcription factor controlling pro-inflammatory gene expression in microglia. Microglial NFκB is a dimer of p50 (*NFκB1)* and p65 (*Rel-A*) [138,139] and is sequestered in the cytoplasm by inhibitor of κB (IκB). Activation of NFκB by inflammatory stimuli is mediated by phosphorylation and ubiquitin-mediated degradation of IκB which disinhibits NFκB, allowing for nuclear translocation to target genes and activation of transcription [140]. For example, microglial activation by the TLR4 agonist, LPS, results in degradation of IκB, translocation of NFκB to the nucleus and activation of κB-containing DNA response elements, including TNFα, IL1β and cyclooxygenase-2 (COX2). NFκB activation by TLR4 signalling results in neuroinflammation and cognitive impairment [141,142].

#### 5.1.1. H_2_O_2_ Activates NFκB Signalling in TLR4-Activated Macrophages

Exogenous application of H_2_O_2_ or induction of H_2_O_2_ release by IL1β or LPS treatment can activate NFκB in multiple cell types, including human myeloid KBM-5 cells, human epithelial-like MCF-7 cells, mouse EL-5 T-cells and macrophages [143,144,145,146]. The source of H_2_O_2_ in response to pro-inflammatory stimulation of MCF-7 cells or macrophages by IL1β or LPS, respectively, is unknown and this could involve activation of H_2_O_2_-generating NOX enzymes, such as NOX4 and DUOX1/2, or the spontaneous reduction of O_2_^•–^ [59]. Multiple studies have shown that H_2_O_2_-mediated NFκB activation results from phosphorylation of IκB tyrosine residues [143,144]. However, this process does not involve the degradation of IκB, which is controlled by IκB kinase (IKK)-mediated serine phosphorylation. H_2_O_2_-mediated activation is, instead, regulated by activation of spleen tyrosine kinase (Syk) [144]. In LPS-stimulated macrophages, the peripheral cousins of microglia, the antioxidant lutein scavenges intracellular O_2_^•–^ and H_2_O_2_ and reduces NFκB activation, whilst TLR4 signalling and MAPK activation remained [146]. Since TLR4 signalling mediates the macrophage and microglial LPS response, which includes MAPK activation, it is suggested that H_2_O_2_ acts downstream of TLR4 signalling, specifically to activate NFκB signalling. Furthermore, pharmacological inhibition or genetic deletion of gp91^phox^ similarly reduces NFκB activation, further suggesting NFκB regulation is redox-sensitive and may be directly linked to NOX2 activity. This NOX2-NFκB interaction is corroborated in a *Pseudomonas aeruginosa* macrophage activation model [147]. Knockdown of gp91^phox^ or application of *N*-acetyl cysteine (the antioxidant, NAC) in infected macrophages reduced NFκB activation levels. Whilst overexpression of gp91^phox^ increased NFκB activation. Further, NFκB may reciprocally regulate NOX2 expression, since its downregulation suppresses gp91^phox^ in the same model. Understanding whether these findings translate to microglia and in response to CNS-relevant stimuli, such as amyloid-β, remains to be determined. Nonetheless, the model of reciprocal expression of NOX2 and NFκB is interesting and could underpin interactions between neuroinflammation and ROS signalling.

#### 5.1.2. There Is a Relationship between NOX Expression and NFκB Activation in Microglia

Specifically, in microglia, LPS treatment results in the activation of mitogen-activated protein kinase kinase kinase 1 (MAP3K1, also known as MEKK1), which is an upstream activator of IKK [148]. Therefore, it is feasible IKK may be activated via this pathway to disinhibit NFκB. However, MAP3K1 regulates many targets involved in oxidative and pro-inflammatory responses, including p38 MAPK, so this may be conjecture [149]. Ethanol-related neurodegeneration is associated with increased microglial NFκB activation and gp91^phox^ expression [150]. Elevated O_2_^•–^ and markers of neurodegeneration were reduced by pharmacological NOX inhibition. Critically, these findings were correlated with human post-mortem brain samples from alcoholic patients, yet the effect of NOX inhibition on NFκB activation in humans was not noted [150].

It is vital to determine pathways of persistent activation in microglia since this underpins the chronic inflammatory state in neurological diseases with an inflammatory aspect [151]. Oxidant production and inflammation are closely linked and it is imperative to properly determine the correct sequence of events since ROS production itself can result from inflammation and NFκB activation via iNOS expression and cytokine-activation of NOX2. Therefore, the coincidence of ROS and NFκB activation in microglia is insufficient to determine a causal relationship. Future studies must determine if there is a genuine interaction or regulation between NFκB and NOX2 in microglia.

### 5.2. ROS Serves to Activate the MAPK Family Resulting in Microglial Activation

The mitogen-activated protein kinase (MAPK) family comprises three subgroups of serine/threonine kinases activated in response to a range of cellular signals, including proinflammatory cytokines, environmental stress and growth factors. The family includes extracellular signal-regulated kinases (ERK1/2), c-Jun N-terminal kinase (JNK) and p38 MAPKs. MAPKs share a common chain of activation mediated by sequential, three-tiered phosphorylation of upstream kinases, i.e., signal transduction is mediated by phosphorylation of a MAPK kinase kinase (MKKK), which in turn activates a MAPK kinase (MKK) which activates the MAPK. MAPKs themselves exert their effects on cellular physiology via myriad effectors, including transcription factors and further kinases [152]. For example, IL1 signalling results in activation of the small G-protein, Ras, which in turn activates MKK6, resulting in activation of p38 MAPK [153].

#### 5.2.1. JNK and p38 MAPK Activation Is Associated with NOX4

ROS may contribute to microglial activation via p38 and JNK activation. Attenuating fluoride-induced oxidative stress in BV-2 microglia with the antioxidant, melatonin, results in reduced JNK activation [154]. Moreover, BV-2 microglia treated with advanced oxidation protein products, a hallmark of oxidative stress, displayed increased NOX4 expression and elevated ROS, along with p38 MAPK and JNK activation [155]. ROS from NOX4 also contribute to activation of the NLRP3 inflammasome, a driver of both amyloid-β [156] and tau [23] pathology. Knockdown of NOX4 by RNAi reduced NLRP3 activation, suggesting a potential target to reduce NLRP3-stimulated pathology in neurodegenerative disease [155]. In a model of spinal cord injury, apocynin, a NOX-inhibitor (see above), reduced activation of p38 MAPK and JNK and expression of TNFα and IL1β [155]. Whilst the ROS generated here are believed to originate from NOX4 expression, the specificity of apocynin for NOX4 has been questioned [157]. Thus, ROS originating from NOX4 activation may be important in activating microglia in response to neuronal damage via stimulation of p38 MAPK and JNK.

#### 5.2.2. ROS of Mitochondrial Origin Contribute to MAPK Activation

Mitochondrial ROS (mROS), independent of NOX-generated ROS, are also involved in the inflammatory response of microglia by activating MAPKs. Specific pharmacological suppression of mROS by mitoTEMPO in LPS-stimulated primary murine microglia resulted in reduced activation of all three MAPKs and limited NFκB nuclear translocation, along with the reduced expression of a battery of pro-inflammatory factors (TNFα, IL1β, IL6, iNOS and Cox-2) [158]. Therefore, MAPK and NFκB activation in microglial cells and the concurrent upregulation of pro-inflammatory mediators is facilitated, at least in part, by mROS. This identifies endogenous ROS derived from mitochondria as critical mediators of the neuroimmune response to LPS, as well as exogenous ROS, i.e., from neuronal damage. However, the selectivity of mitoTEMPO as a mitochondrial-targeted antioxidant has been questioned, especially at higher concentrations [159]. Thorough dose-response analysis, non-selective antioxidant controls and genetic interference of ROS generation from the mitochondria would support the hypothesis that mROS are important in microglial activation, yet such studies are lacking thus far.

## 6. Lipid Droplet-Accumulating Microglia as a Source of OS in Neurodegenerative Disease

The advent of high-resolution transcriptomic techniques, such as scRNAseq, brings the opportunity to study the inherent heterogeneity of microglia and determine different microglial states that are associated with disease progression [10]. One such state that is strongly associated with oxidative stress and neurodegeneration are lipid droplet-accumulating microglia (LDAM).

Lipid droplets (LD) are organelles formed in the endoplasmic reticulum, comprising of a neutral lipid (triacylglycerol and cholesteryl ester) core encapsulated by a phospholipid monolayer, thought to function as a store of energy [160]. In microglia, lipid droplets form in response to innate inflammation, ageing and neurodegeneration and are mediated by canonical pathways of microglial activation, such as p38 MAPK [161,162].

### 6.1. Lipid Droplet Accumulation Is Stimulated by ROS and Correlated with Neuronal Death

In mouse and fly models of neurodegeneration, LD accumulation correlates with the onset of neuronal death, suggesting a crucial role for LDAM in neuronal loss [163]. It was determined that ROS elevation as a result of neuronal mitochondrial dysfunction and JNK activation drives LDAM formation in flies. Antioxidant treatment or human superoxide dismutase 1 (SOD1) expression in flies reduces LD formation and attenuates neurodegeneration [163]. Critically, ROS-stimulated microglial LD formation is mediated, in part, by apolipoproteins and transgenic human AD risk allele, ApoE-ε4, impairs LD formation a fly model [164]. Progranulin (*GRN*) was determined to be a genetic regulator of LDAM formation and loss-of-function mutations in *GRN* are a cause of frontotemporal dementia [162,165]. Interestingly, *GRN*^−/−^ mice display high numbers of LDAM which are ROS-generating and pro-inflammatory, yet defective in phagocytosis [162]. This evidence shows that lipid droplets are an important element of the redox response in microglia and these organelles are associated with AD-risk genes and neuronal death. Further, LDs may be involved in dysregulation of microglial function.

### 6.2. Lipid-Droplet Accumulating Microglia May Represent a Double-Edged Sword in Neurodegeneration

The mechanism of LD accumulation in response to elevated ROS in flies and the perturbation of this mechanism by ApoeE-ε4 indicates LDAM are protective, potentially by sequestering peroxidised lipids [163,164]. However, LDAM are also ROS-generating and pro-inflammatory yet defective in phagocytosis in murine models [162]. This implies LDAM are dysfunctional in neurodegeneration, where they may be unable to clear aggregated protein but would induce oxidative stress and inflammatory cascades. Perhaps sustained oxidative stress, characteristic of neurodegenerative disease, incites a dysregulation in lipid metabolism and compromises the protective nature of LD, leading to release of peroxidised lipid. In this sense, LDAM may underpin a positive feedback loop of stress-mediated ROS generation. It is now imperative to understand what causes protective LDAM to become damaging and translate findings to mammalian models of AD.

## 7. Balancing the Scales—Antioxidant Enzymes Limit Microglial Activation

Many antioxidant proteins are understood to function in the CNS, including classical antioxidants (superoxide dismutase, glutathione reductase, catalase, etc. reviewed in [38]) and more recently TBC, LysM, domain catalytic (TLDc) proteins (oxidation resistance 1, nuclear receptor coactivator 7, Tbc1d24, etc. reviewed in [166]). The function of these redox regulators in microglia is unclear as of yet, however, many of these proteins are linked to inflammation, suggesting functionality in microglia.

### 7.1. Expression of Classical Antioxidant Proteins Are Controlled by Nrf2 in Microglia

Antioxidant genes regulated by nuclear factor (erythroid-derived 2)-like 2 (Nrf2) possess functional roles to limit microglial inflammation. Nrf2 is a basic leucine zipper transcription factor that upregulates genes via interaction with an antioxidant response element (ARE), e.g., genes encoding NADH-quinone oxidoreductase 1 (NQO1) and glutathione-S-transferases (GSTs) [167]. In microglia, Nrf2-deficiency promotes inflammatory marker expression (IL6, Cox2, iNOS) whilst inhibiting anti-inflammatory marker expression (arginase 1, IL4) in response to LPS and MPTP [168,169]. Intracranial injection of the isothiocyanate, sulforaphane, enhanced Nrf2 levels which limited expression of pro-inflammatory microglial markers and conferred neuroprotection [168,169,170]. Microgliosis and sulforaphane neuroprotection were attenuated in Nrf2-deficient mice which confirms a mechanistic link between Nrf2 and immune regulation. Furthermore, heme oxygenase-1 (HO-1), an antioxidant enzyme upregulated by Nrf2, inhibited NOX2 activation in smooth muscle cells and LPS-stimulated macrophages [171,172]. This was mediated by both a reduction in heme availability and biliverdin and carbon monoxide, by-products of heme metabolism by HO-1 which may facilitate attenuation of TLR4 signalling by NOX inhibition [173,174]. Interestingly, deficiency of the microglial housekeeping gene, Cx3cr1, impairs the Nrf2 response in a model of tauopathy and microgliosis is not rescued by sulforaphane indicating the critical role of Cx3cr1 in inflammatory homeostasis [175]. Further studies should investigate the mechanism of Nrf2 regulation by Cx3cr1 and this may uncover modifiable targets for therapeutic intervention in neuroinflammation.

A plethora of plant-derived, quinone-related compounds have been demonstrated to limit microglial inflammation by activation of the Nrf2 pathway and subsequent upregulation of ARE-containing genes, including tiliroside, petatewalide B, emodin and β-lapachone, to name a few [176,177,178,179]. Notably, many experiments examining the role have been conducted in immortalised microglia, e.g., BV2 cells. It is important to validate these responses in vivo, in carefully-isolated primary microglia and iPS-derived microglia to fully understand the role of quinone-derived compounds in neuroinflammatory regulation, especially when considering the deviance of immortalised microglia to that of primary cells [180]. Additionally, it would be more prudent to identify specific, dysregulated targets in disease models and screen for compounds with the potential to modify these disease-relevant targets, as has been eloquently performed by Mendiola et al. [181] (discussed in Section 8.2).

### 7.2. A Role for TLDc Proteins in Neuroinflammation

The TLDc domain is a highly conserved c-terminal domain motif shared by a small family of proteins that protect against oxidative stress in the CNS, such as oxidation resistance 1 (OXR1) and nuclear receptor coactivator 7 (NCOA7) [182]. The mechanism by which TLDc proteins confer stress resistance is unclear, however, specific disruption of the OXR1 TLDc domain in mice is sufficient to cause neurodegeneration and loss-of-function polymorphisms in human TLDc proteins result in a neurodegenerative phenotype [182,183]. Loss of OXR1 results in pronounced microgliosis whilst neuronal OXR1 overexpression delays inflammation, indicating OXR1 regulation of microglial activation in a non-cell-autonomous fashion [184]. Finally, a unique, short-isoform of NCOA7, NCOA7-B or NCOA7-AS, is strongly linked to inflammatory activation, especially by viral-related stimuli such as interferons (IFNs), and functional inhibition of endosome-mediated viral-entry by NCOA7-B has been demonstrated [185]. Furthermore, NCOA7 is induced in epithelial cells in response to rhinovirus infection and LPS-stimulated macrophages, the peripheral myeloid cousins of microglia [186,187]. It is hypothesised that NCOA7-B is a novel interferon-stimulated gene (ISG) as upregulation is stimulated by an array of type I IFNs, including IFNα, IFNα2b and IFNβ1b [188,189,190]. A striking (>60-fold) and robust induction of NCOA7-B occurs in peripheral blood mononuclear cells and macrophages treated with interferon-β-1b (IFNβ1b) or LPS. Furthermore, the expression of this short-isoform in ovarian follicles is regulated intracellularly by MAPKs, p38 and ERK, which are recognised mediators of the pro-inflammatory response in microglia [191]. The function of TLDc proteins in neuroinflammation undoubtedly warrants further investigation, especially by specific genetic manipulation in microglia to aid understanding of the molecular pathways leading to neuroinflammation when TLDc protein expression is disrupted. Understanding how this may link to the oxidative resistance function of TLDc proteins will allow further characterisation of the neuroinflammatory-oxidative stress axis.

Finally, the function of other redox-sensitive enzymes warrants further investigation in microglia, especially those which may have a role in regulating metabolism, ageing and cellular senescence, such as cytochrome b5 reductase 3 (CYB5R3), [192]. Given the close links of ageing, metabolism, lipid droplet accumulation and oxidative stress in microglia, understanding the cross-talk between these cellular regulatory processes is key to developing effective therapeutics and harnessing the protective potential of antioxidant-encoding genes to combat age-related diseases, such as AD.

## 8. Conclusions and Future Directions

### 8.1. Understanding the Molecular Mechanisms of Neuroinflammation Is Key to Developing Effective Antioxidant Therapies

Molecular pathways in microglia contributing to neurodegeneration undoubtedly involve inflammatory-mediated ROS production by activation of NOX. Furthermore, ROS from both NOX and the mitochondria contribute to inflammatory activation of microglia. It is unlikely ROS are the primary trigger of inflammation in Alzheimer’s disease; this is likely mediated by DAMPs associated with toxic protein aggregation. However, it is feasible that ROS act as secondary messengers in microglia to propagate inflammatory states and may contribute to immune dysregulation, leading to persistent and inappropriate inflammation. These pro-inflammatory, pro-oxidant pathways are balanced by antioxidant proteins that limit inflammation. Expression of these proteins by Nrf2 in microglia may represent a pathway that can be modulated to enhance innate defences and combat oxidative stress. The function of other antioxidant proteins in neuroinflammation, such as TLDc proteins, certainly warrants further investigation. Finally, LDAM are important regulators of oxidative stress and a mechanism by which protective inflammation may become dysregulated and damaging in ageing and neurodegeneration.

The question of “what comes first?” is difficult to answer and often lies at the root of disease aetiology. In neuroinflammatory research, the relationship between oxidative stress and inflammation is a central problem. The two are undoubtedly linked; however, it is not clear how ROS interact with glia to elicit an immune response, although this may involve reciprocal regulation between NOX2 and NFκB [147]. The mechanisms leading to ROS production and release by microglia have been explored, especially in CAA where NOX activation likely involves fibrin-related clotting factors and IL13 signalling [104,126]. However, further investigation, especially by cell-specific manipulation of NOX within microglia, is required to fully characterise the inflammatory-induced dysregulation of ROS. The consequences of microglial ROS production on the parenchyma can become pathological, but the impetus for excessive ROS production in a pathological setting is unknown. It hypothesised glia become overzealous in their response to ROS-encouraging pathological stimuli, such as amyloid-β [13]. This likely involves a vicious cycle in which neuroinflammatory signals induce ROS release, which then promotes further deleterious inflammation. To consider this hypothesis, it is necessary to investigate ROS as a key stimulus for inflammation in neurodegeneration. Further, one must consider if the antioxidant capacities or the glial inflammatory brakes are impaired in this neuroinflammatory-oxidative stress cycle. This could involve LDAM as sequesters of excess ROS, which may prevent excessive inflammatory activation or limit ROS concentrations to protect against neuronal damage [164].

Importantly, during the inflammatory response observed in models of neurodegeneration, many molecular mediators of inflammation are activated concurrently. For example, LPS and α-synuclein have a synergistic effect on the upregulation of several key mediators of the inflammatory response, including early activation of PKC, followed quickly by MAPKs and NFκB [84]. Toxic protein species and inflammatory stimuli may work in concert to enhance inflammatory signals via convergent and redundant pathways. For example, the concerted kinase activity of MAPKs and PKC may create a strong pro-oxidant signal by vigorous and persistent phosphorylation of both each other’s activation pathways and NOX2 regulatory subunits, resulting in enhanced and sustained ROS production. Investigating this dynamic interaction between convergent pro-inflammatory pathways, similar to what has been divulged in oncogenic pathways in cancer [193], will be key to understanding inappropriate inflammation in neurodegenerative disease. For effective therapy, it is critical to target these pathways at a suitable upstream point to avoid functional redundancy that may compensate for the perturbation of one target.

### 8.2. At the Forefront of Drug Discovery: ToxSeq Analysis of ROS-Generating Microglia

Recently, crucial steps have been taken in identifying the molecular signature of stress-inducing microglia in neurodegeneration alongside the identification of drugable targets involved in oxidative stress mechanisms. Single-cell RNAseq of ROS-positive microglia (ToxSeq) in an experimental autoimmune encephalomyelitis (EAE) model has identified a unique transcriptional signature in such stress-associated microglia [181]. This signature involved the downregulation of microglial homeostatic genes (*Tmem119, Cx3cr1*) and overexpression of ROS-related genes *Cybb* (p91^phox^), *Cyba* (p22^phox^) and *Nos2* (nitric oxide synthase 2); and pro-inflammatory genes *Il1b, Cd47* and *Prkcd* (protein kinase C-δ).This expression profile concurs with other studies of DAM [10,37,78,124,194]. This genomic-wide approach was coupled with high-throughput drug screening to identify compounds that modulate this specific cell population and alluded to their mechanism of action. Gene network analysis identified upregulation of glutathione transferase activity in ROS-generating microglia, which was rescued by acivin, a compound which inhibits glutathione degradation [181]. Acivin effectively reduced oxidative stress and neuronal death in the EAE model without toxicity to other CNS cell types. The authors were able to characterise the molecular mechanism of acivin, as well as demonstrate improved functional outcome’s in a mammalian model of neurodegeneration.

Discovery of antioxidant/anti-inflammatory drugs that effectively modulate oxidative stress has been hindered by the lack of effective pre-clinical models and understanding of molecular mechanisms that underpin neuroinflammation and ROS production [195]. This new cutting-edge study highlights the requirement for an unbiased selection of relevant targets based on disease mechanisms, whilst also considering the complex transcriptional heterogeneity of microglia [81]. This work, therefore, makes an important contribution to identifying compounds that can modify inflammatory responses and oxidative stress. Looking to the future, if this type of approach can be utilised in a wider range of disease contexts, there is hope that therapies targeting these fundamental pathways can be used to tackle the ever-increasing clinical burden of neurodegenerative disease.
